# Impaired osteoclast homeostasis in the cystatin B-deficient mouse model of progressive myoclonus epilepsy

**DOI:** 10.1016/j.bonr.2015.10.002

**Published:** 2015-11-06

**Authors:** Otto Manninen, Tero Puolakkainen, Jemina Lehto, Elina Harittu, Aki Kallonen, Marko Peura, Tiina Laitala-Leinonen, Outi Kopra, Riku Kiviranta, Anna-Elina Lehesjoki

**Affiliations:** a Folkhälsan Institute of Genetics, 00290 Helsinki, Finland; b Research Program's Unit, Molecular Neurology, University of Helsinki, 00014 Helsinki, Finland; c Neuroscience Center, University of Helsinki, 00014 Helsinki, Finland; d Department of Medicine, University of Turku, 20520 Turku, Finland; e Department of Anatomy, University of Turku, 20520 Turku, Finland; f Department of Physics, University of Helsinki, 00014 Helsinki, Finland

**Keywords:** Cystatin B,, Cathepsin K,, Osteoclast,, Osteogenesis,, Micro-computed tomography,, Progressive myoclonus epilepsy, CSTB, cystatin B, EPM1, progressive myoclonus epilepsy of Unverricht–Lundborg type, μCT, micro-computed tomography

## Abstract

Progressive myoclonus epilepsy of Unverricht–Lundborg type (EPM1) is an autosomal recessively inherited disorder characterized by incapacitating stimulus-sensitive myoclonus and tonic-clonic epileptic seizures with onset at the age of 6 to 16 years. EPM1 patients also exhibit a range of skeletal changes, e.g., thickened frontal cranial bone, arachnodactyly and scoliosis. Mutations in the gene encoding cystatin B (CSTB) underlie EPM1. CSTB is an inhibitor of cysteine cathepsins, including cathepsin K, a key enzyme in bone resorption by osteoclasts. CSTB has previously been shown to protect osteoclasts from experimentally induced apoptosis and to modulate bone resorption in vitro. Nevertheless, its physiological function in bone and the cause of the bone changes in patients remain unknown. Here we used the CSTB-deficient mouse (*Cstb*^−/−^) model of EPM1 to evaluate the contribution of defective CSTB protein function on bone pathology and osteoclast differentiation and function. Micro-computed tomography of hind limbs revealed thicker trabeculae and elevated bone mineral density in the trabecular bone of *Cstb*^−/−^ mice. Histology from *Cstb*^−/−^ mouse bones showed lower osteoclast count and thinner growth plates in long bones. Bone marrow-derived osteoclast cultures revealed lower osteoclast number and size in the *Cstb*^−/−^ group. *Cstb*^−/−^ osteoclasts formed less and smaller resorption pits in an in vitro assay. This impaired resorptive capacity was likely due to a decrease in osteoclast numbers and size. These data imply that the skeletal changes in *Cstb*^−/−^ mice and in EPM1 patients are a result of CSTB deficiency leading to impaired osteoclast formation and consequently compromised resorptive capacity. These results suggest that the role of CSTB in osteoclast homeostasis and modulation of bone metabolism extends beyond cathepsin K regulation.

## Highlights

• μCT reveals changes in trabecular bone of the *Cstb*^−/−^ mouse model of EPM1, compatible with findings in human patients.
• Bone histology in *Cstb*^−/−^ mice shows lower osteoclast number and thinner growth plates in long bones.
• Cultured osteoclasts of *Cstb*^−/−^ mice show decreased size and number of mature osteoclasts with impaired bone resorption.
• Impaired osteoclast formation and resorption are likely to underlie the bone phenotype associated with CSTB deficiency.

## 1 Introduction

Progressive myoclonus epilepsy of Unverricht–Lundborg type (EPM1, OMIM 254800, progressive myoclonus epilepsy type 1) is an autosomal recessively inherited neurodegenerative disorder with onset at the age of 6–16 years. The main symptoms include severely incapacitating action myoclonus, tonic-clonic epileptic seizures and ataxia (Koskiniemi et al., 1974, Kälviäinen et al., 2008). Other central findings include progressive gray and white matter degeneration and changes in cortical excitability (Koskenkorva et al., 2009, Koskenkorva et al., 2012, Danner et al., 2013, Manninen et al., 2013). In addition to the neurological features, EPM1 patients have heterogeneous bone findings (Korja et al., 2007, Suoranta et al., 2012, Danner et al., 2013). They exhibit diffuse thickening of cranial bones in head magnetic resonance imaging (Suoranta et al., 2012). Evaluation of digital head computed tomography (CT) scans have implied osteoporotic bone structure, but, bone mineral density has not been systematically determined (Suoranta et al., 2012). The majority of EPM1 patients also show other skeletal changes, most commonly arachnodactyly, scoliosis, enlarged sinuses, and accessory ossicles of the foot (Suoranta et al., 2012), the etiology of which is currently unknown.

EPM1 is caused by loss-of-function mutations in the *CSTB* gene encoding cystatin B (CSTB), an inhibitor of lysosomal cysteine cathepsins, including cathepsin K (Pennacchio et al., 1996, Lalioti et al., 1997). A mouse model (*Cstb*^−/−^) of the EPM1 disease has been created with targeted disruption of the *Cstb* gene. The mouse model recapitulates the principal symptoms of EPM1, myoclonic seizures and progressive ataxia (Pennacchio et al., 1998). Pathological findings in *Cstb*^−/−^ mice match those in the patients: the mice exhibit progressive loss in brain volume due to neuronal death and consequent white matter atrophy in the cerebrum and cerebellum (Tegelberg et al., 2012, Manninen et al., 2013, Manninen et al., 2014). The earliest pathological finding in *Cstb*^−/−^ mice is striking microglial activation present at postnatal day 14 and preceding the onset of myoclonus and progressive neuronal degeneration from 1 month of age onwards (Tegelberg et al., 2012).

Bone resorbing osteoclasts and bone forming osteoblasts continuously remodel the skeleton to maintain optimal bone structure (Kular et al., 2012). Cathepsin K is one of the fundamental enzymes in osteoclastic bone resorption (Krake et al., 1996, Rantakokko et al., 1996, Littlewood-Evans et al., 1997). CSTB is a known inhibitor of cathepsin K and an in vitro study has shown that it has a role in regulating bone resorption by inhibiting cathepsin K activity and osteoclast apoptosis (Laitala-Leinonen et al., 2006). Overexpression of cathepsin K is known to accelerate the resorption cycle (Morko et al., 2009) and to lead to thickening of cortex in long bone (Morko et al., 2005). The ossification of the calvaria and phalanges is different from rest of the skeleton and Suoranta et al. (2012) proposed that the bone changes in EPM1 patients, most importantly, the thickening of the skull and arachnodactyly, could be mediated through the lack of cathepsin K inhibition, caused by reduced CSTB in cells and this would lead to altered osteoclast function.

Here we utilized the *Cstb*^−/−^ mouse model to evaluate the role of CSTB in bone metabolism and especially in osteoclastic bone resorption by combining micro-computed tomography (μCT) with histological and immunohistochemical analysis of bone tissue and by characterizing bone marrow-derived cultured osteoclasts. Our results show alterations in bone structure of *Cstb*^−/−^ mice and imply impaired formation of mature osteoclasts with consequently compromised resorptive capacity as key phenomena contributing to the altered bone properties.

## 2 Materials & methods

### 2.1 Mouse model

*Cstb*^−/−^ mice (Sv129-^*Cstbtm1Rm*/J^; stock 003486; Jackson Laboratories) between 1 of 4 months of age and wild-type animals of same gender and background as controls were studied. Animals were maintained under a 12 h light/12 h dark cycle, temperature 22 ± 1 °C, and with free access to food and water. The experiments were approved by the Animal Experiment Board of Finland (permits ESLH-2007-05788/Ym-23, ESAVI-2010-07744/Ym-23 and ESAVI/7039/04.10.03/2012) and conducted in agreement with the guidelines set by the European Community Council Directives 86/609/EEC.

### 2.2 Micro-computed tomography

Ex vivo micro-computed tomography (μCT) with SkyScan 1070 μm CT scanner (SkyScan, Kontich, Belgium) was performed on the right hind limb of 3-month-old male mice (*Cstb*^−/−^, n = 5; controls, n = 4). Animals were perfusion fixed with 4% paraformaldehyde and hind limbs post fixed for 24 h, then stored immersed in 70% EtOH. The fixed hind limbs were inserted into a sealed tubular plastic imaging holder (manufactured in-house). The following parameters were used for the scanning: voxel resolution 20 μm; X-ray tube potential 70 kVp; current 200 μA; and integration time 3900 ms. The object was rotated in 0.45° steps (total 182.45°). Cross-sectional image reconstruction using Nrecon software version 1.4 (SkyScan, Kontich, Belgium) was performed with the following parameters: misalignment < 3, ring artifact reduction 11, beam hardening correction 95% and intensity gap 0.005–0.150. Analysis and modeling were performed using CTan version 1.4.4 (SkyScan, Kontich, Belgium). Cross-sectional images were binarized using an attenuation coefficient range of 0.005 to 0.150. In trabecular bone, a volume of interest (VOI) excluding the cortical bone was defined at the metaphysis of the tibia, starting 20 layers (400 μm) below the lowest point of the growth plate and covering 50 layers (1000 μm). Cortical bone analysis was done from diaphysis of the femurs, with VOI starting 200 layers (4000 μm) above the growth plate and covering 100 layers (2000 μm). Finally, the 3D, 2D and bone mineral density results were calculated into numerical data, with low and high threshold values set to 35 and 250 respectively, after which the data were analyzed.

### 2.3 Bone histology

#### 2.3.1 Samples

Bone histology was performed on the hind limbs of *Cstb*^−/−^ and control mice, age 1 month (*Cstb*^−/−^, n = 13; controls; n = 13) and 4 months (*Cstb*^−/−^, n = 13; controls, n = 13) Animals were perfusion fixed with 4% paraformaldehyde, hind limbs removed and post fixed for 24 h, then stored immersed in 70% EtOH. Samples were prepared from distal femurs and proximal tibias, and the bones were decalcified in 5% formic acid, embedded in paraffin and cut into 5 μm-thick sections for mounting on microscopy slides. Femur length was also determined using a micrometer (*Cstb*^−/−^, n = 5; controls, n = 3).

#### 2.3.2 Quantification of bone morphology

Bone morphology was analyzed using hind limb samples from 1-month-old animals (*Cstb*^−/−^, n = 8; controls, n = 8). Quantitative analysis of the thickness of the growth plate and its proliferative- and hypertrophic zones was performed as previously described (Nurmio et al., 2011). Briefly, bone sections were stained with hematoxylin and quantified using Leica QWin Pro analysis system (Leica Microsystems GmbH, Wetzlar, Germany). The number of mature tartrate-resistant acid phosphatase (TRACP, stained with the leukocyte acid phosphatase kit (Sigma Aldrich, St. Louis, Missouri, USA)) positive multinucleated osteoclasts was counted as described previously (Laitala-Leinonen et al., 2006) from samples taken from 1-month-old (*Cstb*^−/−^, n = 13; controls, n = 13) and 4-month-old animals (*Cstb*^−/−^, n = 13; controls; n = 13).

### 2.4 Characterization of cultured osteoclasts

#### 2.4.1 Bone marrow-derived osteoclast cultures

Osteoclasts were cultured as described previously (Laitala-Leinonen et al., 2006). In brief, animals were euthanized with CO_2_ and decapitated. Bone marrow was harvested from both femurs and tibias, and homogenized to a single cell suspension. The cell suspension was then allowed to adhere for 4 h, after which the non-adhering cells, including the macrophage precursors, were plated at 100,000 cells/well on 96-well plates in α-MEM. The culture medium was supplemented with macrophage colony stimulating factor (M-CSF) 20 ng/ml (RnD systems, Minneapolis, MN, USA), which induces the hematopoietic stem cells to differentiate into macrophages. Every 48 h half of the media was replaced and receptor activator of nuclear factor kappa-B ligand (RANKL, Peprotech, Offenbach, Germany) 100 ng/ml was added to facilitate osteoclast differentiation.

#### 2.4.2 TRACP staining of cultured osteoclasts

First, osteoclast morphology and number was determined from cultures.

The osteoclasts were cultured for 6 days on RANKL, after which they were fixed with 4% PFA and stained with the leukocyte acid phosphatase kit (Sigma Aldrich, St. Louis, Missouri, USA). For determining the number and the area of mature osteoclasts, the samples were imaged using Olympus ScanR system (Olympus GmbH, Hamburg, Germany), a modular epifluorescence microscope designed for fully automated image acquisition. The number of mature osteoclasts, defined a TRACP-positive multinuclear (three or more nuclei) cells, was counted from 22 wells for both *Cstb*^−/−^ and wild type controls (cells pooled from 3 mice per group in three independent experiments). The size of the osteoclasts was quantified from 9 wells of both *Cstb*^−/−^ and controls (cells pooled from 4 mice per group). The quantification was done using ImageJ version 1.48 ROI-manager plugin (http://rsbweb.nih.gov/).

#### 2.4.3 Quantitative real-time PCR

Total RNA was isolated from osteoclasts (*Cstb*^−/−^ mice n = 3; control mice, n = 3, all male, cells pooled) cultured for 6 days on RANKL using Qiagen RNAeasy kit (Qiagen, Heiden, Germany) following the instructions of the manufacturer. Qiagen RT^2^ Profiler PCR Array osteogenesis kit (Qiagen, Hilden, Germany) was then used to assess gene expression differences in *Cstb*^−/−^ osteoclasts compared to controls (136 ng of RNA used for both groups). The results were analyzed using Qiagen RT^2^ Profiler data analysis web portal (Qiagen, Hilden, Germany).

#### 2.4.4 Cathepsin K assay in cultured osteoclasts

Osteoclasts were cultured for 5 days in the presence of RANKL, after which the activity of cathepsin K was assessed using the Magic Red Cathepsin K assay Kit (Immunochemistry Technologies, Bloomington, MN, USA) according to the manufacturer's instructions. The fluorescence was measured from 8 sample wells of both *Cstb*^−/−^ and controls (cells pooled from 4 mice per group) using Pherastar FS plate reader (BMG Labtech, Ortenberg, Germany). A reference number of cells per well was counted from a 10 × field obtained using an Incyte Zoom (Essen Bioscience, Ann Arbor, MI, USA) system. The number of cells per field was determined with ImageJ version 1.48 using Analyze Particles plugin.

#### 2.4.5 Osteoclast pit formation assay

For assaying resorption pit formation, osteoclasts were cultured on 96-well Corning osteo-assay surface plates (Corning, New York, NY, USA) that contain a synthetic bone coating. Culturing and analysis were performed according to manufacturer's recommendations. In brief, 300,000 cells/well were plated in α-MEM including M-CSF 20 ng/ml (RnD systems, Minneapolis, MN, USA). Every 48 h half of the media was replaced and RANKL was added (100 ng/ml, Peprotech, Offenbach, Germany). Cells were cultured for 7 days with RANKL to allow osteoclasts to resorb the synthetic bone coating. The cells were then fixed with 4% PFA, washed with PBS and removed from the wells with a 5 min treatment with 10% sodium hypochlorite, thus exposing the resorption pits for microscopy.

According to the manufacturer recommendations, the formed resorption pits were visualized by counterstaining the synthetic bone coating with 1% toluidine blue (Sigma Aldrich, St. Louis, Missouri, USA). As the resorption pits formed by osteoclasts extend to the bottom of the wells, toluidine blue staining makes resorption pits readily visible as clear regions against the dark stained background of the synthetic bone coating. The stained samples (*Cstb*^−/−^, 10 wells; controls, 10 wells, cells pooled from 4 mice per genotype) were imaged with Olympus ScanR (Olympus GmbH, Hamburg, Germany). A matrix of 6 × 8 images per well at 10 × magnification were obtained. For counting the number and size of the resorption pits, proprietary ScanR software (version 1.3.0.3, Olympus GmbH, Hamburg, Germany) was used. The edges of the resorption pits were detected using the edge-detection feature in the software that is based on large image intensity difference between the dark synthetic bone and the light resorption pit. From these detected objects, positive hits for resorption pits were first filtered for object size to exclude false positive hits caused by well edges. Then a staining intensity threshold was used to exclude black staining artifacts left from the toluidine blue staining.

### 2.5 Statistics

Statistical analyses were done with Student's t-test using Microsoft Excel for Mac 2011 (Microsoft, Redmond, Washington, USA) with a p-value < 0.05 considered significant.

## 3 Results

### 3.1 Altered bone structure and osteoclast homeostasis in *Cstb*^−/−^ mice

To investigate the contribution of deficient CSTB function on bone metabolism we first analyzed the bone structure in hind limbs of 3 month old *Cstb*^−/−^ mice with μCT. Analysis of the trabecular bone mineral density (BMD) revealed significantly higher BMD in *Cstb*^−/−^ mice (0.339 ± 0.02 vs. control 0.243 ± 0.01, p = 0.001). The difference in BMD of cortical bone was not significant (*Cstb*^−/−^ 0.208 ± 0.01 vs. control 0.189 ± 0.06). Analysis of bone structure revealed changes in trabecular bone (Table 1), including increased bone volume (bone volume/tissue volume) and trabecular thickness. These data indicate that the lack of CSTB in the *Cstb*^−/−^ mouse results in increased bone mass especially in the trabecular bone. As trabecular number and trabecular separation did not show statistically significant difference, it would appear that the change in bone volume is due to increased trabecular thickness.

**Fig. 1:**
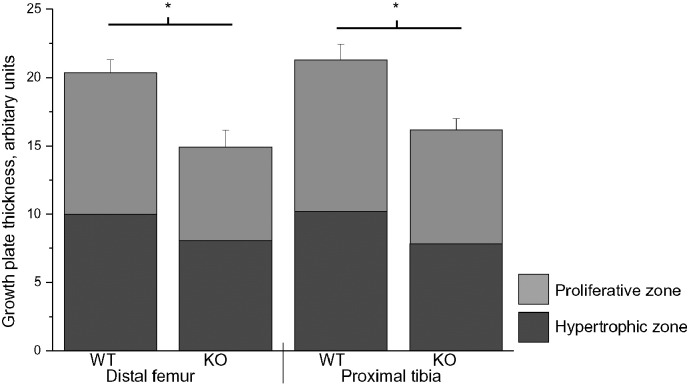
Growth plates are thinner in *Cstb*^−/−^ mice.

As many of the bone changes observed in the EPM1 patients, such as arachnodactyly and assessory ossicles of the foot, are connected to skeletal growth, we next measured growth plate thickness from fixed and decalcified hind limb sections. The *Cstb*^−/−^ mice had significantly thinner growth plates in both distal femurs and proximal tibias already at 1 month compared to controls (Fig. 1), giving further evidence for CSTB being required for normal skeletal growth. However, as no differences between *Cstb*^−/−^ and wild-type animals were seen in thickness of proliferative or hypertrophic zones, it appears that the structure of the growth plates remains unaffected. Nevertheless, even thought *Cstb*^−/−^ mice have been reported to be smaller and weighting less, no difference in femur length was observed (*Cstb*^−/−^ 14.9 mm ± 1.7 vs. control 16.3 ± 0.0).

Quantification of bone morphology showed significantly thinner growth plates in both distal femurs and proximal tibias of *Cstb*^−/−^ mice (n = 8) when compared to controls (n = 8), but no difference in thickness of profilerative or hypertrophic zones. *p < 0.05.

**Fig. 2:**
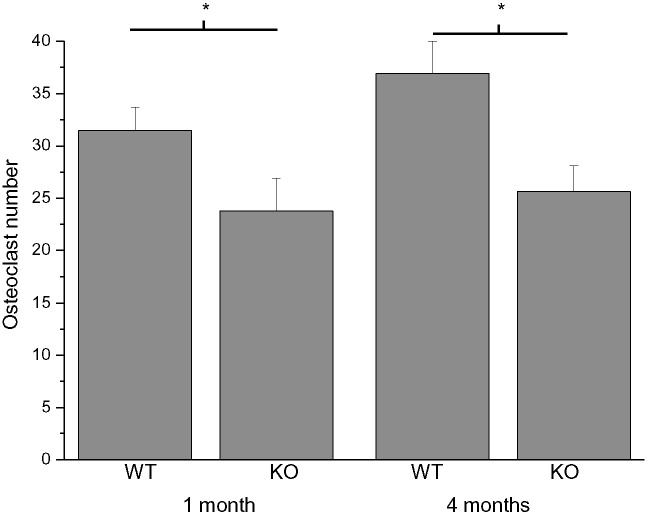
Histology shows decreased osteoclast numbers and increased staining intensity of TRACP 5b and cathepsin K in *Cstb*^−/−^ mice.

To further investigate the underlying causes for the changes in bone morphology observed in μCT and analysis of growth plate thickness, the number of osteoclasts was first quantified from TRAPC-stained sections of fixed and decalcified hind limb bone tissue. The analysis revealed a significantly lower number of osteoclasts per microscopy field in *Cstb*^−/−^ mice (Fig. 2). The difference was observed in both 1-month-old and 4-month-old animals both in distal femurs and proximal tibias. There was no difference in the number of osteoclasts per microscopy field between tibias and femurs.

A.) Quantification of mature osteoclast numbers per microscopy view from TRAPC-stained decalcified bone section from distal femurs and proximal tibias of 1-month-old (*Cstb*^−/−^, n = 13; controls, n = 13) and 4-month-old animals (*Cstb*^−/−^, n = 13; controls, n = 13) shows a significant decrease in osteoclasts numbers in *Cstb*^−/−^ mice when compared to controls.

### 3.2 Lower number and impaired bone resorption of cultured *Cstb*^−/−^ osteoclasts

**Fig. 3:**
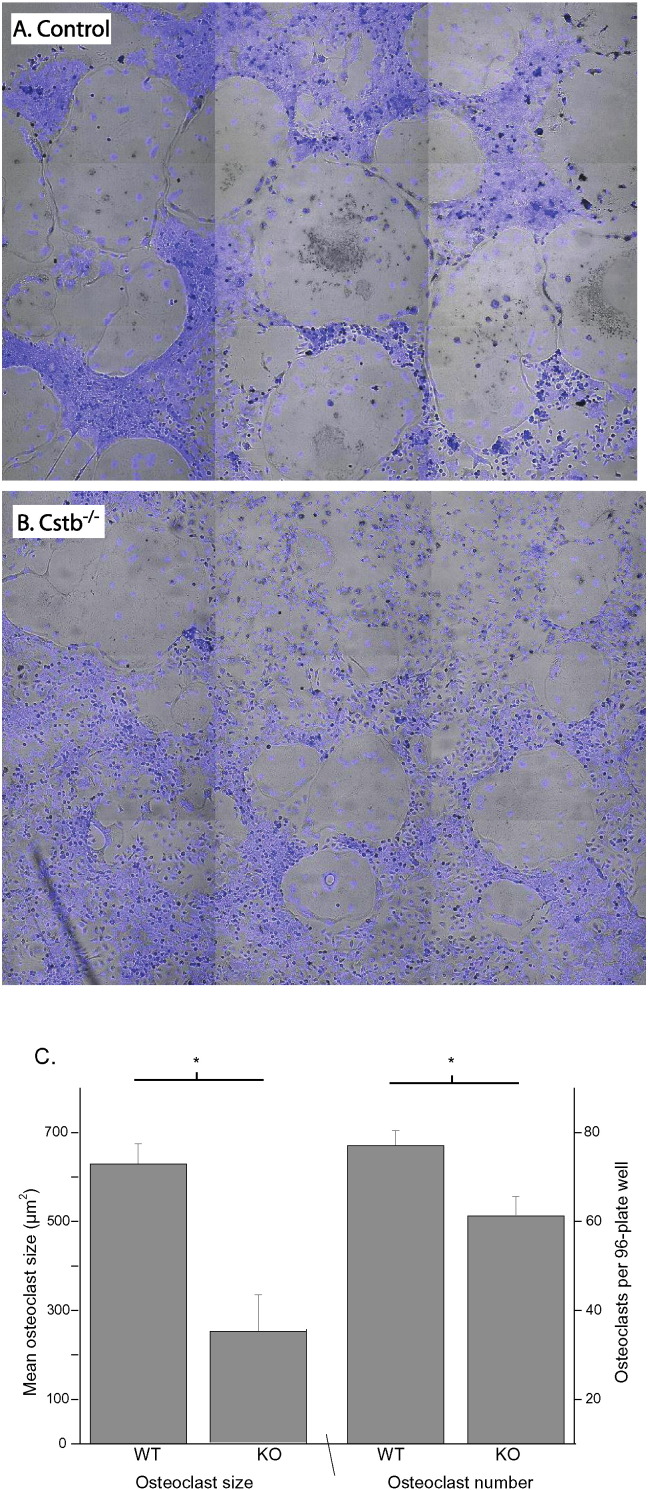
The number and size of bone marrow-derived cultured osteoclasts is lower in *Cstb*^−/−^ than control mice.

As analysis of bone tissue suggested an osteoclast phenotype in *Cstb*^−/−^ mice, we further characterized the osteoclasts in bone marrow-derived osteoclast cultures. In line with the lower osteoclast count in bone tissue, culturing non-adherent bone marrow cells from *Cstb*^−/−^ mice resulted in lower number of multinucleated TRACP positive osteoclasts than cells from control mice (Fig. 3). The size of cultured osteoclast was significantly smaller in *Cstb*^−/−^ compared to control cultures (Fig. 3).

A. and B.) A representative 3 × 3 matrix of microscopy fields (10 × magnification) from bone marrow-derived osteoclast cultures. For identifying mature multinucleated osteoclasts, cultures have been stained for TRACP (black stain on light microscopy) and Hoechst (overlaid blue fluorescence), and show lower osteoclast number and size in the *Cstb*^−/−^ group. C.) Quantification of osteoclast cultures show significant decrease in both the size (*Cstb*^−/−^, n = 9 wells; controls, n = 9 wells) and the number (*Cstb*^−/−^, n = 22 wells; controls, n = 22 wells) of *Cstb*^−/−^ osteoclasts (*p < 0.05).

To reveal possible causes for the lower osteoclast number in *Cstb*^−/−^ mice we characterized the composition of the progenitor cell population in bone marrow as well as the viability and presence of apoptosis in cultured osteoclasts (Supplementary data). A smaller hematopoietic precursor population was hypothesized to lead to changes in the number of mature osteoclasts, but the number of hematopoietic precursors was actually slightly higher in the *Cstb*^−/−^ mice. No indication for changes in viability or apoptosis of cultured osteoclasts was observed.

Finally, qPCR-based gene expression profiling of cultured osteoclasts was performed to investigate whether gene expression changes could implicate possible causes for the difference in osteoclast numbers. Expressions of 84 genes related to osteogenesis and bone mineral metabolism were assayed (Supplementary Table 1). Of these, 21 were upregulated with a fold change of ≥ 1.5 and 17 were downregulated with a fold change of ≤− 1.5 (Table 2). The function of the genes showing altered expression was related to cell growth, differentiation and osteoclastic function.

Gene expression profiling showed a 1.87 fold upregulation of cathepsin K gene expression (Table 2) in line with the higher staining intensity of cathepsin K (Supplementary data) in bone tissue. To investigate this further, we first measured cathepsin K activity from cultured osteoclasts using a fluorescence-based assay. The enzyme activity normalized to the number of cells was not significantly different between *Cstb*^−/−^ and control osteoclasts (*Cstb*^−/−^ 0.82 vs. control 1.00 fluorescence units, t-test p = 0.15).

**Fig. 4:**
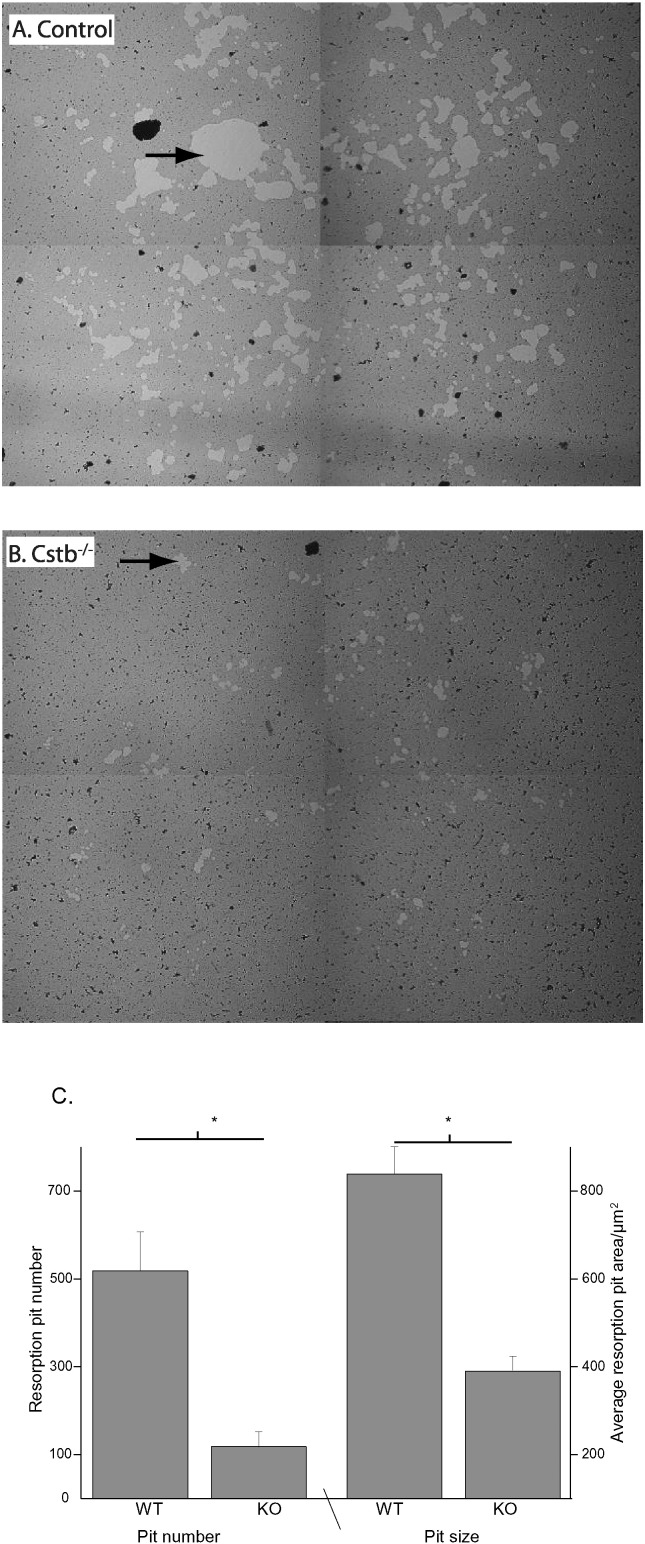
Osteoclast pit formation assay indicates lower resorption by *Cstb*^−/−^ osteoclasts.

We then studied the resorption capacity of osteoclasts that were cultured on synthetic bone coating and assayed the number of resorption pits formed. The number and size of the formed resorption pits was found to be significantly lower in the *Cstb*^−/−^ group compared to control cultures (Fig. 4). As resorption capacity of individual cells seems not to be compromised, as indicated by staining of the cathepsin K and of TRACP 5b in bone tissue (Supplementary data), the results suggest that the reduced bone resorption in the *Cstb*^−/−^ cultures results from the lower osteoclast count.

A. and B.) A representative 2 × 2 matrix of microscopy fields (10 × magnification) obtained from a resorption pit formation assay performed with bone marrow-derived osteoclast cultures. The assay demonstrates that smaller and fewer resorption pits are formed by *Cstb*^−/−^ osteoclasts. Osteoclasts cultured on synthetic bone coating form resorption pits that extend to the bottom of the well. When the synthetic bone is counterstained with toluidine blue (dark stain), the pits are observed as light areas (indicated by black arrows). The black staining artifacts are caused by toluidine blue. C.) Quantification of the resorption pits (*Cstb*^−/−^ n = 10 wells; controls, n = 10 wells) shows that *Cstb*^−/−^ osteoclasts form significantly less and smaller pits compared to controls. *p < 0.05.

## 4 Discussion and conclusions

We investigated the cause for the skeletal changes observed in EPM1 patients and the role of CSTB in bone tissue by characterizing the bone and osteoclast phenotype in the *Cstb*^−/−^ mouse model of EPM1. This model recapitulates well the key neurological and neuropathological features of the human disease, (Pennacchio et al., 1998, Kälviäinen et al., 2008, Manninen et al., 2013, Tegelberg et al., 2012) but the bone phenotype of *Cstb*^−/−^ mice had not been studied before. Analysis of bone morphology and composition with μCT revealed thicker and more heavily mineralized trabecular bone in the *Cstb*^−/−^ mice, whereas no differences in cortical bone structure were seen. In EPM1 patients, the diffuse skull thickening affects also the trabecular bone (Korja et al., 2007, Suoranta et al., 2012). While the implied osteoporotic changes in calvarial bones of EPM1 patients could indicate decreased BMD, but, due to the retrospective study setup, no BMD measurements, or quantification of structural changes were performed (Suoranta et al., 2012).

The other bone changes reported in EPM1 patients, e.g. scoliosis, arachnodactyly and the presence of accessory ossicles in the feet, are linked to skeletal formation (Suoranta et al., 2012). However, no obvious macroscopic morphological bone changes were identified in *Cstb*^−/−^ mice. While osteoblast cultures did not indicate apparent differences in bone formation between *Cstb*^−/−^ and control mice (Supplementary data), analysis of growth plate thickness revealed them to be significantly thinner in *Cstb*^−/−^ mice. Taken together these data suggest that changes in bone growth and remodeling contribute to the observed skeletal changes in the EPM1 patients. The contribution of medication, especially of phenytoin that has been reported to cause calvarial thickening (Kattan, 1970) and of the reduced mobility due to incapacitating myoclonus of the EPM1 patients to the observed bone changes has previously been discussed (Suoranta et al., 2012). However, as the bone phenotype is present already in young mice and in in vitro systems, our results in *Cstb*^−/−^ mice indicate that the bone changes in EPM1 patients are a direct result of deficient CSTB function.

The cell cultures from *Cstb*^−/−^ mice have not indicated an osteoblast phenotype in *Cstb*^−/−^ mice that would contribute to the observed morphological change (Supplementary data), however bone histology and bone marrow-derived osteoclastic cultures revealed fewer osteoclasts of smaller size in *Cstb*^−/−^ mice compared to control mice. This could be caused by lower progenitor cell population, lower viability, failure in differentiation and/or maturation or increased death of osteoclasts in *Cstb*^−/−^ mice. However, the bone marrow progenitor cell population was slightly larger in *Cstb*^−/−^ animals and no significant differences in cell viability between *Cstb*^−/−^ and control mice were observed. Furthermore, even if previous reports have shown that CSTB is capable of inhibiting osteoclast apoptosis and regulate bone resorption in vitro (Laitala-Leinonen et al., 2006), and that cathepsin K modulates osteoclastic apoptosis and senescence (Chen et al., 2007, Wilson et al., 2009), no differences were seen in the apoptotic death of osteoclasts between *Cstb*^−/−^ and control mice. The significantly smaller size of cultured *Cstb*^−/−^ osteoclasts, however, implies a failure in the fusion and/or differentiation of the precursor cells to mature osteoclast. Interestingly, qPCR-based expression profiling of genes relevant to osteogenesis revealed altered expression of several genes related especially to osteoclastic differentiation and function. All seven bone morphogenic proteins (BMPs), known to stimulate osteoclast differentiation and survival (Itoh et al., 2001, Okamoto et al., 2006, Wutzl et al., 2006) in the assay were downregulated in *Cstb*^−/−^ cultures. Of these, BMP3 showed 8-fold downregulation. Activin A receptor 1, which relays BMP signals and is known to play an important role in osteoclast differentiation (Fuller et al., 2000), was downregulated 1.5-fold. Genes related to the resorptive function such as cathepsin K and matrix metallopeptidase 9 (MMP9), which digest the collagen matrix in bone (Reponen et al., 1994), were upregulated. However, whether these changes in gene expression are responsible for the bone changes observed in vivo, or merely reflect the phenotype of the cultured cells, remains to be investigated.

CSTB is a known inhibitor of cathepsin K and immunohistochemical analysis of osteoclasts in distal femurs and proximal tibias indicated higher cathepsin K expression in *Cstb*^−/−^ mice (Supplementary data). Also, CSTB has been reported suppressing resorption by osteoclasts in vitro (Laitala-Leinonen et al., 2006). Contrary to these results, the pit formation assay showed that the overall resorption capacity, at least in vitro, is significantly compromised in the *Cstb*^−/−^ osteoclasts. Therefore, increased TRACP 5b and cathepsin K staining in bone tissue (Supplementary data), combined with elevated cathepsin K gene expression in *Cstb*^−/−^ osteoclasts could be an indication of a compensatory mechanism to the inadequate resorptive function. Nevertheless, resorption pit formation assay has been shown to be a good quantitative measure of osteoclast resorption activity (Fuller et al., 1994), and decrease in osteoclast numbers has previously been linked with an increase in trabecular thickness and BMD (Boyce et al., 2014). As resorptive function of osteoclasts is closely linked to the cell size and number (Xing et al., 2012), the impaired resorptive function suggests that the changes in bone morphology, e.g., more heavily mineralized bone and the thicker trabeculae result from lower osteoclast number and size. Therefore, we feel safe to conclude that the changes in bone morphology in the *Cstb*^−/−^ mice, and of EPM1 patients, result from inadequate resorptive capacity of osteoclasts due to their decreased number and size, which is likely to be caused by impaired formation of mature osteoclasts.

In light of the recent findings in *Cstb*^−/−^ mice linking CSTB function to inflammation (Maher et al., 2014, Okuneva et al., 2015) it is possible that inflammatory mechanisms contribute to the altered bone homeostasis. Interestingly, epigenetic processes were recently linked to differentiation of osteoclasts, the defect of which resulted in an osteoclast and bone phenotype resembling that of *Cstb*^−/−^ mice (Nishikawa et al., 2015). Therefore, as CSTB is known to have a nuclear localization and function (Alakurtti et al., 2005, Ceru et al., 2010), similar mechanisms could be involved in EPM1 as well. While the mechanism by which lack of CSTB leads to observed skeletal changes remains to be identified, our results nevertheless suggest that the role of CSTB in osteoclasts is more complex than simply acting as an inhibitor of cathepsin K.

## Figures and Tables

**Table 1:** Quantified morphology derived from the μCT of trabecular bone in hind limbs of 3 month old *Cstb*^−/−^ and control mice.

	*Cstb*^−/−^	Control
Trabecular bone mineral density	0.34 ± 0.02	0.24 ± 0.01⁎
Cortical bone mineral density	0.21 ± 0.01	0.19 ± 0.06
Percent bone volume (%)	24.7 ± 1.18	18.3 ± 0.61⁎
Trabecular thickness (mm)	0.17 ± 0.01	0.14 ± 0.01⁎
Structure model index	1.30 ± 0.35	0.26 ± 0.18⁎
Trabecular number (1/mm)	1.43 ± 0.09	1.33 ± 0.03
Trabecular separation (mm)	0.54 ± 0.01	0.56 ± 0.01

⁎ p < 0.05.

**Table 2:** Genes showing at least 1.5-fold expression changes in *Cstb*^−/−^ osteoclasts compared to controls.

Gene	Fold upregulation	Gene explanation
*Comp*	4.65	Cartilage oligomeric matrix protein
*Col10a1*	4.25	Collagen, type X, alpha 1
*Mmp9*	3.96	Matrix metallopeptidase 9
*Mmp8*	3.52	Matrix metallopeptidase 8
*Gli1*	3.40	GLI-Kruppel family member GLI1
*Cd36*	3.24	CD36 antigen
*Bmpr1b*	2.51	Bone morphogenetic protein receptor, type 1B
*Itga2*	2.32	Integrin, alpha 2
*Egf*	2.17	Epidermal growth factor
*Col2a1*	1.93	Collagen, type II, alpha 1
*Fgf2*	1.93	Fibroblast growth factor 2
*Itgam*	1.89	Integrin, alpha M
*Ctsk*	1.87	Cathepsin K
*Tnfsf11*	1.76	Tumor necrosis factor (ligand) superfamily, member 11
*Col4a1*	1.69	Collagen, type IV, alpha 1
*Spp1*	1.69	Secreted phosphoprotein 1
*Tgfb1*	1.61	Transforming growth factor, beta 1
*Icam1*	1.59	Intercellular adhesion molecule 1
*Tgfbr1*	1.55	Transforming growth factor, beta receptor 1
*Smad1*	1.53	SMAD family member 1
*Vegfb*	1.51	Vascular endothelial growth factor B


Gene	Fold downregulation	Gene explanation
*Bmp3*	− 8.02	Bone morphogenetic protein 3
*Bglap*	− 4.21	Bone gamma carboxyglutamate protein
*Bmp4*	− 4.12	Bone morphogenetic protein 4
*Bmp1*	− 2.54	Bone morphogenetic protein 1
*Ihh*	− 2.16	Indian hedgehog
*Fgfr2*	− 2.11	Fibroblast growth factor receptor 2
*Gdf10*	− 2.08	Growth differentiation factor 10
*Sost*	− 1.99	Sclerostin
*Bmp2*	− 1.90	Bone morphogenetic protein 2
*Fgf1*	− 1.87	Fibroblast growth factor 1
*Pdgfa*	− 1.81	Platelet-derived growth factor alpha polypeptide
*Csf3*	− 1.75	Colony stimulating factor 3
*Nog*	− 1.69	Noggin
*Runx2*	− 1.64	Runt related transcription factor 2
*Sox9*	− 1.62	SRY (sex determining region Y)-box 9
*Acvr1*	− 1.50	Activin A receptor, type I
*Dlx5*	− 1.50	Distal-less homeobox 5
