# Supplementary material for: Impaired osteoclast homeostasis in the cystatin B-deficient mouse model of progressive myoclonus epilepsy
Source: Bone Rep. 2015 Nov 6;3:76–82. doi: 10.1016/j.bonr.2015.10.002 (PMC5365244; doi:10.1016/j.bonr.2015.10.002)
Supplement: Supplementary file 1 — Supplementary material. [file mmc1.docx]

**SUPPLEMENTARY DATA**

**Bone immunohistochemistry**

The enzymatic properties of the osteoclasts were characterized by immunohistochemical staining for TRACP 5b and cathepsin K (Supplementary Figure 1).The staining intensities were quantified from hind limb bone samples from 1-month-old animals (*Cstb^-/-^*, n=10; controls, n=10) as described previously (Nurmio et al. 2011). In brief, staining was done using Cathepsin K monoclonal antibody in 1:600 dilution (Acris Antibodies GmbH, Hiddenhausen, Germany) and tartrate-resistant acid phosphatase 5b (TRACP 5b) polyconal antibody in 1:500 dilution (Halleen et al. 1998) and detected with 1:50 TRITC donkey anti-mouse antibody (Jackson ImmunoResearch Laboratories, Inc., West Grove, USA). A staining control for specificity on both antibodies was performed by substituting primary antibodies with mouse normal serum. Both the number of immunopositive cells and the intensity of the staining were quantified using Leica QWin Pro analysis system (Leica Microsystems GmbH, Wetzlar, Germany).

Quantification of staining intensity implied higher presence of both TRACP 5b and cathepsin K per osteoclast in *Cstb^-/-^* mice (Supplementary Figure 1).

**Supplementary Figure 1. Quantification suggests increased staining intensity of TRACP 5b and cathepsin K in *Cstb^-/-^* mice.**

A. and B.) Representative images of a double staining in osteoclast for TRACP 5b (blue stain) and cathepsin K (dark brown stain) in fixed and decalcified bone section from a control and a *Cstb^-/-^* mouse. C.) Quantification of the staining intensities (*Cstb^-/-^*, n=10; controls, n=10) from sections separately stained against either TRACP 5b or cathepsin K revealed significantly higher staining intensity in *Cstb^-/-^* compared to control osteoclasts. The staining intensity in controls is shown as 100% (* p<0.05).

**Fluorescence activated cell sorting**

Fluoresence activated cell sorting (FACS) was performed from 3-month-old male mice (*Cstb^-/-^* n=5; control, n=3). In brief, animals were euthanized with CO_2_ and decapitated. Bone marrow was harvested from both femurs and tibias, and homogenized to a single cell suspension in a-MEM and 1 000 000 cells were used for stainings. Briefly, after treating with blocker (CD16/CD32, eBioscience), the cells were incubated with an antibody cocktail for lineage depletion (CD3e, CD11b, CD45R (B220), Ly-6G and Ly-6C (Gr-1), TER-119 (Ly-76), BD Pharmingen, San Jose, California, USA) for 30 min on ice. After washing the cells with PBS, they were stained with streptavidin conjugated FITC stain and antibodies for Sca-1 and cKit (BD Pharmingen) for 30 min on ice. After washing with PBS the stained cells were were counted using the FACScalibur system (BD Biosciences, Franklin Lakes, New Jersey, USA). The data was analysed with Flowing Software (v. 2.5.1) (BD Biosciences, Franklin Lakes, New Jersey, USA).

The number of hematopoetic precursors (lineage negative, Sca-1 and cKit positive cells) was slightly higher in the *Cstb^-/-^* mice (*Cstb^-/-^* 395 ± 56 cells per 100 000 counted vs. control 247 ± 46 cells per 100 000 counted; p=0.03).

**Cell viability assay**

Osteoclasts (*Cstb^-/-^* mice n=3; control mice, n=3, cells pooled) were cultured for 48 h, after which CellTiter 96® AQ_ueous_ One Solution Cell Proliferation Assay (Promega, Fitchburg, Wisconsin, USA) was used for determining possible differences in cell viability between the genotypes. The assay was performed according to manufacturer’s instructions. For each well, 40 ul of the reagent was added and incubated for 2h at 37^o^C, after which the absorbance at 490 nm was measured using a Wallac Victor2 plate reader (Perkin Elmer, Waltham, Massachusetts, USA).

The results indicated no change in cell proliferation (*Cstb^-/-^* 0.335 ± 0.007 absorbance units vs. control 0.328 ± 0.006 absorbance units; p=0.12).

**Terminal deoxynucleotidyl transferase dUTP nick end labeling (TUNEL)**

Osteoclasts (*Cstb^-/-^* mice n=3; control mice, n=3, cells pooled) were cultured 6 days on RANKL, after which the amount of apoptotic osteoclasts was assessed using TACS Blue Label TUNEL-based Apoptosis Detection Assay (R&D Biosystems, Minneapolis, Minnesota, USA). The staining was performed according to manufacture instructions, and a positive control was prepared from cells of the control group by treating the cells with a DNA digesting reagent included provided in the kit. The positive control was stained with the same protocol as *Cstb^-/-^* and control osteoclasts. Microscopy did not reveal osteoclasts with TUNEL-positive nuclei in *Cstb^-/-^* and control groups, whereas the positive control showed intense staining.

**Supplementary Figure 2. TUNEL-staining shows no indication of increased osteoclast apoptosis in *Cstb^-/-^* cells**

A. and B.) TUNEL staining for apoptotic nuclei indicates no difference between cultured *Cstb^-/-^* and control osteoclasts. C.) Positive control; the TUNEL-positive nuclei are identified with blue staining.

| Supplementary Table 1.  Gene expression changes in *Cstb^-/-^* osteoclasts compared to controls. Genes with a fold change of at least 1.5 are depicted in bold text. | | | | |
| --- | --- | --- | --- | --- |
| Gene | **Fold change** | | | **Gene explanation** |
| *Acvr1* | | **-1,50** | **Activin A receptor, type I*** | |
| *Ahsg* | | -1,03 | alpha-2-HS-glycoprotein | |
| *Alpl* | | -1,30 | alkaline phosphatase in liver/bone/kidney | |
| *Anxa5* | | 1,15 | annexin A5 | |
| *Bglap* | | **-4,21** | **bone gamma carboxyglutamate protein*** | |
| *Bgn* | | -1,01 | biglycan | |
| *Bmp1* | | **-2,54** | **bone morphogenetic protein 1*** | |
| *Bmp2* | | **-1,90** | **bone morphogenetic protein 2*** | |
| *Bmp3* | | **-8,02** | **bone morphogenetic protein 3*** | |
| *Bmp4* | | **-4,12** | **bone morphogenetic protein 4*** | |
| *Bmp5* | | -1,28 | bone morphogenetic protein 5 | |
| *Bmp6* | | -1,18 | bone morphogenetic protein 6 | |
| *Bmp7* | | -1,12 | bone morphogenetic protein 7 | |
| *Bmpr1a* | | -1,18 | bone morphogenetic protein receptor, type 1A | |
| *Bmpr1b* | | **2,51** | **bone morphogenetic protein receptor, type 1B*** | |
| *Bmpr2* | | 1,44 | bone morphogenetic protein receptor, type II | |
| *Cd36* | | **3,24** | **CD36 antigen*** | |
| *Cdh11* | | -1,07 | cadherin 11 | |
| *Chrd* | | 1,25 | chordin | |
| *Col10a1* | | **4,25** | **collagen, type X, alpha 1*** | |
| *Col14a1* | | 1,25 | collagen, type XIV, alpha 1 | |
| *Col1a1* | | -1,02 | collagen, type I, alpha 1 | |
| *Col1a2* | | 1,02 | collagen, type I, alpha 2 | |
| *Col2a1* | | **1,93** | **collagen, type II, alpha 1*** | |
| *Col3a1* | | 1,05 | collagen, type III, alpha 1 | |
| *Col4a1* | | **1,69** | **collagen, type IV, alpha 1*** | |
| *Col5a1* | | 1,18 | collagen, type V, alpha 1 | |
| *Comp* | | **4,65** | cartilage oligomeric matrix protein | |
| *Csf1* | | 1,47 | colony stimulating factor 1 | |
| *Csf2* | | 1,41 | colony stimulating factor 2 | |
| *Csf3* | | **-1,75** | **colony stimulating factor 3*** | |
| *Ctsk* | | **1,87** | **cathepsin K*** | |
| *Dlx5* | | **-1,50** | **distal-less homeobox 5*** | |
| *Egf* | | **2,17** | **epidermal growth factor*** | |
| *Fgf1* | | **-1,87** | **fibroblast growth factor 1*** | |
| *Fgf2* | | **1,93** | **fibroblast growth factor 2*** | |
| *Fgfr1* | | -1,32 | fibroblast growth factor receptor 1 | |
| *Fgfr2* | | **-2,11** | **fibroblast growth factor receptor 2*** | |
| *Flt1* | | 1,25 | FMS-like tyrosine kinase 1 | |
| *Fn1* | | -1,10 | fibronectin 1 | |
| *Gdf10* | | **-2,08** | **growth differentiation factor 10*** | |
| *Gli1* | | **3,40** | **GLI-Kruppel family member GLI1*** | |
| *Icam1* | | **1,59** | **intercellular adhesion molecule 1*** | |
| *Igf1* | | 1,36 | insulin-like growth factor 1 | |
| *Igf1r* | | 1,25 | insulin-like growth factor 1 receptor | |
| *Ihh* | | **-2,16** | **Indian hedgehog*** | |
| *Itga2* | | **2,32** | **integrin, alpha 2*** | |
| *Itga2b* | | 1,17 | integrin, alpha 2b | |
| *Itga3* | | -1,30 | integrin, alpha 3 | |
| *Itgam* | | **1,89** | **integrin, alpha M*** | |
| *Itgav* | | 1,44 | integrin, alpha V | |
| *Itgb1* | | 1,11 | integrin, beta 1 | |
| *Mmp10* | | -1,12 | matrix metallopeptidase 10 | |
| *Mmp2* | | 1,28 | matrix metallopeptidase 2 | |
| *Mmp8* | | **3,52** | **matrix metallopeptidase 8*** | |
| *Mmp9* | | **3,96** | **matrix metallopeptidase 9*** | |
| *Nfkb1* | | 1,41 | nuclear factor of kappa light polypeptide gene enhancer in B cells 1 | |
| *Nog* | | **-1,69** | **noggin*** | |
| *Pdgfa* | | **-1,81** | **platelet-derived growth factor alpha polypeptide*** | |
| *Phex* | | -1,22 | phosphate regulating endopeptidase homolog, X-linked | |
| *Runx2* | | **-1,64** | **runt related transcription factor 2** | |
| *Serpinh1* | | -1,08 | serine (or cysteine) peptidase inhibitor, clade H, member 1 | |
| *Smad1* | | **1,53** | **SMAD family member 1*** | |
| *Smad2* | | 1,36 | SMAD family member 2 | |
| *Smad3* | | 1,19 | SMAD family member 3 | |
| *Smad4* | | 1,14 | SMAD family member 4 | |
| *Smad5* | | -1,05 | SMAD family member 5 | |
| *Sost* | | **-1,99** | **sclerostin*** | |
| *Sox9* | | **-1,62** | **SRY (sex determining region Y)-box 9*** | |
| *Sp7* | | -1,42 | Sp7 transcription factor 7 | |
| *Spp1* | | **1,69** | **secreted phosphoprotein 1*** | |
| *Tgfb1* | | **1,61** | **transforming growth factor, beta 1*** | |
| *Tgfb2* | | 1,28 | transforming growth factor, beta 2 | |
| *Tgfb3* | | -1,04 | transforming growth factor, beta 3 | |
| *Tgfbr1* | | **1,55** | **transforming growth factor, beta receptor 1*** | |
| *Tgfbr2* | | 1,33 | transforming growth factor, beta receptor 2 | |
| *Tgfbr3* | | -1,29 | transforming growth factor, beta receptor 3 | |
| *Tnf* | | 1,10 | tumor necrosis factor | |
| *Tnfsf11* | | **1,76** | **tumor necrosis factor (ligand) superfamily, member 11*** | |
| *Twist1* | | -1,43 | twist basic helix-loop-helix transcription factor 1 | |
| *Vcam1* | | 1,00 | vascular cell adhesion molecule 1 | |
| *Vdr* | | -1,06 | vitamin D receptor | |
| *Vegfa* | | 1,03 | vascular endothelial growth factor A | |
| *Vegfb* | | **1,51** | **vascular endothelial growth factor B*** | |

**Osteoblast culture**

Osteoblasts cultures were performed as described previously (Morko et al. 2009). In short, the mice were euthanized with CO_2_, and femurs and tibias dissected. Consequently, the diaphysis was exposed and bone marrow harvested and homogenized to a single cell suspension. The cells were then seeded into culture flasks at density of 10^6^/cm^2^. The cells were cultured for 7 days in a-MEM media with 10 mM Na-β-glycerophosphate and 50 μg/ml ascorbate-2-phosphate and with 10^-8^ M dexamethasone (Sigma, St. Louis, Missouri, USA). The adherent cells were then scraped, washed and plated at 10 000 cells/well onto 24 well plates and cultured for another 14 days, without dexamethasone. Cultures were then fixed with 4% PFA, and the osteoblasts were stained for morphological assessment with leukocyte alkaline phosphatase kit (Sigma, St. Louis, Missouri, USA) and with von Kossa protocol, using 2% sodium nitrate and 2.5% sodium thiosulfate solutions (Sigma, St. Louis, Missouri, USA). This staining did not indicate apparent differences between osteoblast cultures derived from *Cstb^-/-^* and control mice. (Supplementary Figure 3).

**Supplementary Figure 3. Von Kossa stained osteoblast cultures**

Von Kossa staining of cultured osteoblasts indicated no difference in bone formation between cultured control (A.) and *Cstb^-/-^* (B.) osteoblasts.

**Reference**

Halleen, J.M., Hentunen, T.A., Karp, M., Käkönen, S.M., Pettersson, K., Väänänen, H.K., 1998. Characterization of serum tartrate-resistant acid phosphatase and development of a direct two-site immunoassay. J. Bone Miner. Res. 13 (4), 683–687 (Apr).
